# COVID-19 Transmission Due to Delta Variant in New York City Public Schools From October to December 2021

**DOI:** 10.1001/jamanetworkopen.2022.13276

**Published:** 2022-05-23

**Authors:** Jay K. Varma, Cara Feldkamp, Mariana Alexander, Emily Norman, Tracy Agerton, Rindcy Davis, Theodore Long

**Affiliations:** 1 Department of Population Health Sciences, Weill Cornell Medicine, New York, New York; 2 Test and Trace Corps, New York City Health and Hospitals, New York, New York; 3 New York City Department of Health and Mental Hygiene, New York, New York

## Abstract

This cohort study assesses COVID-19 prevention measures against the Delta variant by measuring the secondary attack rate for cases in students, teachers, and staff of New York City public schools from October to December 2021 and analyzing the likely direction of transmission for secondary cases.

## Introduction

The New York City public school system is the largest in the US, with more than 1600 schools and more than 1 million enrolled students. For the 2021-2022 school year, COVID-19 safety measures included mandatory vaccination for all adults; daily health screening; universal face coverings; improved indoor air quality using windows, portable air purifiers, and/or central air filtration; testing of unvaccinated persons; and quarantine for unvaccinated contacts.^1^ There was substantial concern that the Delta variant, which caused a surge in July to August 2021, would disrupt the school year. To assess the effectiveness of COVID-19 prevention measures against the Delta variant, we measured the secondary attack rate for COVID-19 cases diagnosed in students, teachers, and staff from October 10 to December 5, 2021, and analyzed the likely direction of transmission for secondary cases, stratified by vaccination status.

## Methods

New York City agencies receive reports of COVID-19 cases through multiple data systems and identify school-based contacts in collaboration with teachers and administrators (eAppendix in the Supplement). Close contacts who are unvaccinated and symptomatic are monitored for COVID-19 symptoms for 10 days after their exposure date and encouraged to undergo testing 3 to 5 days after exposure. We restricted the analysis to index cases identified from October 10 to December 5, 2021, owing to changing contact definitions and vaccination data at the beginning of the school year and the emergence of Omicron variant community transmission during the week of December 5. Data on close contacts with an exposure date were analyzed through December 27, 2021. We calculated the secondary attack rate as the number of school-based close contacts who developed COVID-19 divided by the total number of school-based close contacts, expressed as a percentage. No individual written informed consent was obtained for this program. No institutional review board was consulted for this study because all data were collected by government agencies as part of public health surveillance activities to control an acute health emergency (45 CFR §46) and this program was implemented as part of a legally-required COVID-19 health and safety policy from the New York City Department of Education and legally required public health data collection by the New York City Health Department. (In addition, New York state law mandates that all local school districts collect and report data about COVID-19 cases in the school community and that local health departments collect data about all COVID-19 cases occurring in their jurisdiction.) The study followed the Strengthening the Reporting of Observational Studies in Epidemiology (STROBE) reporting guidelines. All analyses were conducted with R, version 4.0.3 (R Group for Statistical Computing). Additional details are in the eAppendix in the Supplement.

## Results

Of 91 173 individuals with COVID-19 and close contacts, 74 411 (81.6%) were students, with a median age of 11 years (IQR, 8-13 years), and 16 762 (18.4%) were staff, with a median age of 40 years (IQR, 32-48 years). Among 9604 individuals with primary cases of COVID-19, we identified 80 980 school-based close contacts exposed to 4381 individuals with COVID-19; 589 individuals with secondary cases of COVID-19 were exposed to 474 individuals with index cases of COVID-19, for a secondary attack rate of 0.7% (Table). The secondary attack rate was 0.6% (n = 107) for 18 682 fully vaccinated contacts and 0.9% (n = 394) for 43 304 unvaccinated contacts. Among both vaccinated and unvaccinated contacts who developed cases of COVID-19, the individual with the index case of COVID-19 was most often (323 of 477 [67.7%]) a student. The ratio of primary to secondary cases of COVID-19 was 16:1.

**Table.** COVID-19 Secondary Attack Rate and Direction of Transmission by Vaccination Status Among New York City Public School Close Contacts of Index Cases From October 10 to December 5, 2021

Characteristic	No./total No. (%)		
	All	Fully vaccinated	Unvaccinated
Total No. of close contacts	80 980	18 682	43 304
Close contacts who tested positive within 14 d of exposure, No. (%)	589 (0.7)	107 (0.6)	394 (0.9)
Close contacts for whom direction of infection was inferred	477/589 (81.0)	103/107 (96.3)	345/394 (87.6)
Staff to staff	34/477 (7.1)	29/103 (28.2)	1/345 (0.3)
Staff to student	120/477 (25.2)	11/103 (10.7)	103/345 (29.9)
Student to staff	56/477 (11.7)	48/103 (46.6)	2/345 (0.6)
Student to student	267/477 (56.0)	15/103 (14.6)	239/345 (69.3)

## Discussion

From October to December 2021, the secondary attack rate of COVID-19 in New York City public schools was 0.7%. The secondary attack rate helps answer the policy-relevant question: If a person with COVID-19 is present in school, how likely is it that they will transmit infection to other people in that school? Restricting the analysis to persons exposed inside a school provides a direct, albeit imperfect, way to measure the effectiveness of in-school prevention measures. In fall 2021, the secondary attack rate was comparable to that in fall 2020 in New York City, lower than that in multiple North Carolina schools when Delta was dominant, and lower than that in households.^2,3,4^

This analysis is subject to important limitations. The rate could be an overestimate because there is no accurate way to classify whether contacts were infected from the school-based individual with an index case of COVID-19 that triggered quarantine or from a different source inside or outside school. The rate could be an underestimate because contacts are not universally tested and monitoring excludes fully vaccinated, asymptomatic persons, consistent with Centers for Disease Control and Prevention guidance during this period. Even if there was extreme underascertainment of COVID-19 among contacts, multiplying the overall secondary attack rate by 3 (for a maximum plausible estimate of 2.2%) means that only about 2 in 100 school-based contacts were infected. Definitive conclusions should not be drawn about vaccine effectiveness because neither vaccination status nor testing for all exposed contacts was fully complete.

## Notes

Supplement. **eAppendix.** Supplemental Methods
