# Supplementary material for: COVID-19 Transmission Due to Delta Variant in New York City Public Schools From October to December 2021
Source: JAMA Netw Open. 2022 May 23;5(5):e2213276. doi: 10.1001/jamanetworkopen.2022.13276 (PMC9127551; doi:10.1001/jamanetworkopen.2022.13276)
Supplement: Supplement. — eAppendix. Supplemental Methods [file jamanetwopen-e2213276-s001.pdf]

Supplementary Online Content

Varma JK, Feldkamp C, Alexander M, et al. COVID-19 transmission due to Delta variant in New York City public schools from October to December 2021. *JAMA Netw Open.* 2022;5(5):e2213276. doi:10.1001/jamanetworkopen.2022.13276

eAppendix. Supplemental Methods

This supplementary material has been provided by the authors to give readers additional information about their work.

© 2022 Varma JK et al. *JAMA Network Open.*

eAppendix. Supplemental Methods

Case and Contact Investigations

The Situation Room is a New York City government entity that coordinates COVID-19 investigations and control measures in public schools. It is staffed by personnel from the New York City Health + Hospitals Test & Trace Corps and the Departments of Education, Health and Mental Hygiene, and Buildings. Reports of COVID-19 cases associated with schools are received in the Situation Room through three sources. First, as mandated by law, clinical laboratories are required to report to the Department of Health and Mental Hygiene information about any city resident with a positive viral diagnostic test (antigen or molecular). New, laboratory-confirmed cases (or their parent/guardian) are interviewed and asked about any association with a school, and school-associated cases are notified to the Situation Room. Second, all school-affiliated persons are required and actively encouraged to notify their school if they are diagnosed with COVID-19, and schools notified the Situation Room about these cases; such reports are necessary if the person is not a New York City resident. Third, viral diagnostic testing is performed on a 10% sample of unvaccinated persons in each school once per week, with results reported directly from clinical laboratories to the Situation Room. This sampling method was implemented consistent with CDC guidance.

The New York City Test & Trace Corps conducts daily text messages or every-other-day calls to persons identified as close contacts for 10 days after their date of exposure, except for those that are fully vaccinated and asymptomatic at the time they are identified. Persons being monitored are asked about symptoms of COVID-19 for 10 days after their date of exposure.

© 2022 Varma JK et al. *JAMA Network Open.*

Definition and Management of Close Contacts and Contacts who Became Cases

A close contact is defined as a person who was less than six feet away from an infected person (laboratory-confirmed or clinical diagnosis) for a cumulative total of 10 minutes or more over a 24-hour period. In the K–12 indoor classroom setting where mask use can be observed, the close contact definition changed on September 27, 2021 to align with CDC guidance (https://www.cdc.gov/coronavirus/2019-ncov/daily-life-coping/K-12-infographic.html) to exclude students who were between 3 to 6 feet of an infected student (laboratory-confirmed or a clinical diagnosis) if both the infected student and the exposed student(s) correctly and consistently wore well-fitting masks the entire time. This exception did not apply to adults (e.g., teachers, staff, or other adults) in the indoor classroom setting. Any student who shared a classroom with an adult case, regardless of physical distance and mask use, was considered a close contact, resulting in entire classrooms being quarantined for an adult case. Starting October 20, 2021, this policy for adult cases was changed: a close contact was a person who was less than 6 feet from an adult case for a cumulative total of 10 minutes or more over a 24-hour period.

If a close contact is unvaccinated, they are required to stay at home for 10 days after their date of exposure; those who test negative on day 5 or later may return to school on day 8 after their date of exposure if they remain asymptomatic. If a close contact is vaccinated, they are permitted to remain in school as long as they do not develop symptoms of COVID-19; these contacts are encouraged to get tested 3-5 days after their exposure. Further details are available at: https://www.schools.nyc.gov/school-life/health-and-wellness/covid-information/health-and-safety-in-our-schools

© 2022 Varma JK et al. *JAMA Network Open.*

A contact was defined as a new case if they tested positive for COVID-19 by an authorized test (confirmed case) within 14 days of their date of exposure in school. Due to limitations in data systems regarding reports of negative COVID-19 tests, we were not able to measure accurately the proportion of contacts who underwent testing.

Fully Vaccinated and Unvaccinated Definitions

A person was defined as fully vaccinated if they reported to the Department of Education that they received at least two doses of a COVID-19 mRNA-containing vaccine or one dose of the Janssen vaccine at least 14 days before their exposure. A person was defined as unvaccinated if they reported to the Department of Education that they did not receive any doses of a WHO-approved vaccine or if the person was under the age of 5 as they would not be eligible for this vaccination.

Although the school year started on September 13, 2021, data on vaccination status was not available for routine analysis until October 10, 2021. At the time of this analysis, we did not have information on the vaccination status for 18,994 (23.46%) close contacts. The number of close contacts with unknown status decreased over time, particularly as mandates in and out of school went into effect, but there was still a notable lag between when students age 5-11 became approved for vaccines and when students were required to report their vaccination status. We suspect that a substantial number of people with unknown status have been vaccinated but have not yet reported their status to their school, because the secondary attack rate among people with unknown vaccination status (88 contacts became cases; 0.46%) was lower than the rate among vaccinated people (0.57%) and much lower than the rate among unvaccinated people (0.91%).

© 2022 Varma JK et al. *JAMA Network Open.*

Direction of Transmission

For all contacts who tested positive or became ill, we reviewed the case and contact interviews to determine whether we could plausibly infer the direction of transmission (who infected whom). Scientific consensus is that people who develop COVID-19 infection do not generally test positive by molecular or antigen tests or develop symptoms until at least 3 days after exposure. We excluded contacts from the direction of transmission analysis if they tested positive or developed symptoms less than 3 days after exposure, because we cannot plausibly attribute their infection to in-school or out-of-school exposure. Of the instances where we could infer directionality of transmission, we classified them according to the following scheme: staff to staff; staff to student; student to staff; student to student.

Period of Analysis

Given the changing definitions of close contacts and the emergence of community Omicron transmission in mid-December, we restricted our period of analysis to cases identified from October 10 – December 5, 2021 and close contacts identified from October 10 – December 27, 2021, which gave close contacts two weeks to become cases and time for cases to be reported.

Data Management

To determine how many close contacts became cases, we matched a database of close contacts to a database of unique cases. We then removed people from the matched file who were listed as their own close contact (i.e., close contacts who were listed as being exposed to their own case number) and removed instances when there were duplicate rows of the same close contact being

© 2022 Varma JK et al. *JAMA Network Open.*

exposed to the same case. To create the right window of time, we narrowed the list of matches down to instances where the reported date of symptoms and/or positive test minus the date of exposure was less than or equal to 14 days; we called these "validated matches." The secondary attack rate was calculated as the number of validated matches divided by the number of de-duplicated close contacts, expressed as a percentage. For the directionality of transmission analysis, we further narrowed the list of validated matches to include only people who became cases three or more days after exposure and had only one exposure. Some close contacts have been exposed to multiple cases; when that happens, we cannot make an inference about which case infected the close contact.

© 2022 Varma JK et al. *JAMA Network Open.*
